# Probiotics for children with asthma: a systematic review and meta-analysis

**DOI:** 10.3389/fped.2025.1577152

**Published:** 2025-04-24

**Authors:** Yang Liu, Yuxiao Zhang, Yingna Li, Xiaohu Zhang, Liang Xie, Hanmin Liu

**Affiliations:** ^1^ Department of Pediatric Pulmonology and Immunology, West China Second University Hospital, Sichuan University, Chengdu, China; ^2^ Key Laboratory of Birth Defects and Related Diseases of Women and Children, Sichuan University, Ministry of Education, Chengdu, China; ^3^ NHC Key Laboratory of Chronobiology, Sichuan University, Chengdu, China; ^4^ The Joint Laboratory for Lung Development and Related Diseases of West China Second University Hospital, Sichuan University and School of Life Sciences of Fudan University, West China Institute of Women and Children’s Health, West China Second University Hospital, Sichuan University, Chengdu, China; ^5^ Sichuan University-The Chinese University of Hong Kong Joint Laboratory for Reproductive Medicine, West China Second University Hospital, Sichuan University, Chengdu, China; ^6^ Development and Related Diseases of Women and Children Key Laboratory of Sichuan Province, West China Second University Hospital, Sichuan University, Chengdu, Sichuan, China

**Keywords:** probiotics, asthma, children, meta-analysis, pulmonary function

## Abstract

**Background:**

Asthma is a common chronic inflammatory disease affecting children worldwide. While probiotics have been proposed as a potential therapy, their efficacy in pediatric asthma management remains controversial.

**Methods:**

A systematic search of PubMed, Web of Science, Embase, Cochrane Central Register of Controlled Trials (CENTRAL) and clinicaltrials.gov was conducted to identify randomized controlled trials (RCTs) from 2014 to 2024 evaluating probiotic interventions in children with asthma. Primary outcomes included asthma exacerbation rates and predicted FEV1%. The risk of bias was assessed using Cochrane guidelines.

**Results:**

Out of 1,361 articles, eight RCTs involving 902 participants were included. Meta-analysis showed probiotics significantly reduced acute asthma episodes with risk ratio of 0.38 (95% CI: 0.26–0.56, *p* < 0.00001) and improved FEV1/FVC ratios (MD = 5.70, 95% CI: 1.93–9.47, *p* < 0.003) compared to the control group. Neither FEV1 levels nor school attendance showed significant changes.

**Conclusion:**

Probiotic supplementation may reduce asthma exacerbations and improve pulmonary function in pediatric asthma. However, heterogeneity across studies suggests the need for further research to determine optimal strains, dosages, and treatment durations. This review establishes groundwork for research and practice by exploring microbial interventions in childhood airway disorders.

**Systematic Review Registration:**

https://www.crd.york.ac.uk/PROSPERO/view/CRD42024607569, identifier (CRD42024607569).

## 1 Introduction

Children with asthma experience chronic inflammation, leading to various respiratory symptoms that significantly impact their daily lives. These manifestations include episodic breathing difficulties, characterized by wheezing and chest tightness, which can substantially affect their physical activity and school attendance. It is a common long-term condition affecting children worldwide, impacting their quality of life and posing a significant burden on healthcare systems. Recent World Health Organization (WHO) surveillance data reveals the substantial global burden of asthma, with the condition affecting an estimated 262 million individuals worldwide as of 2019, contributing to over 455,000 deaths annually (1). Over the past two decades, pediatric asthma prevalence has generally shown an upward trend (2). Despite inhaled corticosteroids providing symptom control, persistent cases may still develop serious complications and lung dysfunction (3, 4). Treatment options remain limited for young patients due to several constraints: adverse endocrine reactions, expensive biological therapies, and age restrictions (5). There is a continuous pursuit of additional and complementary treatments to further improve outcomes for children with asthma.

Live microorganisms that benefit host health when adequately administered, known as probiotics (6), are increasingly being considered as an adjunct therapy for respiratory and allergic disease management, highlighting the potential treatment for children with asthma. Emerging studies have shown that the intestinal microbiota composition of children with asthma is significantly different from that of healthy children (7), with the diversity of intestinal microbiota decreased, the number of bifidobacterium decreased, Th1 cytokines (IFN-g and TNF-a) down-regulated, and Th2-type cytokines (IL-4, IL-5) and Th17-type cytokine (IL-17A)s up-regulated (8). Dysbiosis can affect the gut-lung axis, triggering inflammatory cascades, while supplementing probiotics or synbiotics can restore symbiosis and manipulate immune responses (9–11). Evidence suggests that microbial therapy can effectively alleviate asthmatic symptoms (12, 13), but there are also controversial results (14, 15). Heterogeneity may arise from different strains of probiotics used, as well as the timing of supplementation during pregnancy and after birth, leading to differences in analysis outcomes. Some studies have indicated that prenatal and perinatal supplementation has little preventive effect on asthma (16, 17). Therefore, this study focuses on children with diagnosed asthma and specifically examines research from the past decade (2014–2024) to understand recent advancements in the use of probiotics for managing pediatric asthma, with an emphasis on their potential benefits in symptom management, reduction of asthma attack frequency and severity, and overall impact on children's quality of life.

## 2 Materials and method

We structured our research methodology following the latest Preferred Reporting Items for Systematic Reviews and Meta-Analyses (PRISMA) guidelines for systematic review and meta-analysis (18). To ensure transparency and reproducibility, we pre-registered our study protocol in the International Prospective Register of Systematic Reviews database (PROSPERO, Registration ID: CRD42024607569).

### 2.1 Search strategy

A systematic search spanning 2014–2024 was conducted across the following electronic databases, including PubMed, Web of Science, Embase, Cochrane Central Register of Controlled Trials (CENTRAL) and clinicaltrials.gov. This time frame was selected to capture the most recent advances in probiotic research and clinical trial methodologies. The literature search was restricted to studies published in English due to resource limitations for translating non-English articles. Duplicate records were identified and removed using EndNote reference management software, followed by manual verification by two independent reviewers. Using search terms mainly including probiotics, asthma, children, etc. (Supplementary File 1), multiple independent reviewers completed the screening by November 2024, 28.

### 2.2 Selection criteria

Studies were considered eligible upon fulfilling five criteria: (1) population: children diagnosed with asthma aged 0–18 years old; (2) intervention: probiotics; (3) comparison: either placebo or control group; (4) out-comes: reported in rate of asthma exacerbations and predicted percentage of forced expiratory volume in first second (FEV1%); (5) study design: randomized controlled trials (RCTs). Ineligible studies: (1) non-asthma conditions: individuals with other respiratory conditions that mimic asthma, e.g., chronic obstructive pulmonary disease, cystic fibrosis, or bronchiolitis; (2) lack of clear diagnosis: studies where the diagnosis of asthma is not clearly defined or confirmed will be excluded to ensure that the population under review is homogeneous; (3) studies with inaccessible full texts or datasets, even after attempt to contact the authors; (4) duplicate publications or studies with overlapping data; (5) conference abstract or papers with insufficient methodological details. To assess inter-reviewer agreement during the full-text screening phase, Cohen's kappa statistic was calculated. The kappa value was 0.79, indicating substantial agreement between reviewers. This reflects the robustness and reliability of the study selection process.

### 2.3 Quality assessment

RCTs were evaluated using the Cochrane Risk of Bias 2 (RoB 2) tool, which examines five domains of potential bias: the randomization process, adherence to intended interventions, outcome data completeness, outcome measurement, and result reporting selectivity. The assessment was conducted independently by multiple reviewers. To evaluate the certainty of evidence, we applied the GRADE (Grading of Recommendations Assessment, Development and Evaluation) framework. This framework assesses evidence quality across five domains: study design, inconsistency, indirectness, imprecision, and publication bias.

### 2.4 Data extraction

Literature screening and data extraction were conducted independently by two researchers with cross-validation. Disagreements were resolved through discussion or third-party consultation. The selection process began with a review of titles to exclude irrelevant literature, followed by an assessment of abstracts and full texts to determine study inclusion. Data extraction covered publication metadata (author, year, country), participant demographics (sample size, age, gender), intervention specifics (probiotic type, dosage, duration), and clinical endpoints (exacerbation rates, FEV1%).

### 2.5 Statistical analysis

Analysis was performed using RevMan 5.4. Efficacy was primarily assessed through relative risk (RR) and 95% confidence intervals (95% CI) for enumeration data. Heterogeneity among studies was assessed using the Q-Cochran test and the *I*^2^ statistic. Considering the limitations of the Q-Cochran test, such as its low sensitivity in detecting inconsistency, the *I*^2^ statistic was primarily used to quantify heterogeneity. The interpretation of *I*^2^ value followed standard thresholds: 0%–40% indicate low heterogeneity (possibly negligible), 30%–60% moderate heterogeneity, 50%–90% substantial heterogeneity, and 75%–100% considerable heterogeneity. Model selection was based on the heterogeneity assessment: fixed-effect for homogeneous studies (*p* > 0.1, *I*^2^ < 40%), while random-effects when significant heterogeneity was detected (*p* < 0.1, *I*^2^ > 40%). Source of heterogeneity were explored through subgroup analysis. Publication bias was evaluated using funnel plots.

## 3 Results

### 3.1 Literature search

**Figure 1:**
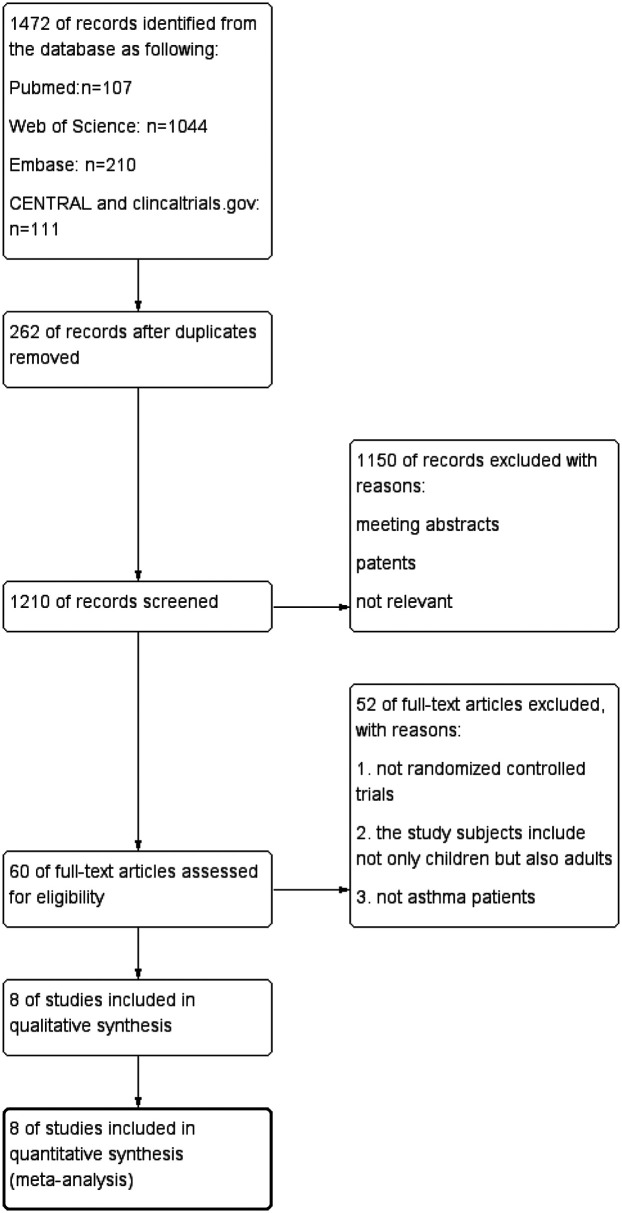
Flow chart of the stepwise procedure for study selection.

Databases searches yield 1,472 records: 107 from PubMed, 1,044 articles were found in the Web of Science, 210 from Embase, 106 from CENTRAL and 5 from clinical.gov. After deduplication (*n* = 262), 1,210 articles were screened. Of these, 1,150 were excluded as conference papers, patent, or irrelevant studies. Full-text review of the remaining 60 articles identified 8 eligible studies, encompassing 902 participants for final analysis (19–26). Figure 1 illustrates the selection process.

The eight articles ultimately included in our review were all RCTs sourced from SCI-indexed journals. The studies focused on distinct aspects of probiotic influence: two explored the link between probiotics and asthma exacerbation rates, two examined the impact on FEV1 levels, and three assessed the relationship with FEV1/FVC ratios. Additionally, two studies investigated probiotic efficacy on the Childhood Asthma Control Test (CAT) scores, and another two looked into the association with blood immune-related factors. However, due to the variation in detection methods and the heterogeneity in reported units and data presentation across studies, a meta-analysis was not feasible on CAT and immunologic indicators. These articles spanned multiple countries and regions, featured diverse probiotics or their combinations, and included a range of dosages and intervention periods. Table 1 summarized the characteristics of each study.

**Table 1:** A summary of characeristics of included studies.

ID	Author	Year	Country or Region	Type of probiotics or synbiotic	Dose and Duration	Duration and follow-up	Age (years) of placebo group	Age (years) of probiotic group	Total number	Number of placebo group	Number of probiotic group	Gender (M/F) of placebo group	Gender (M/F) of probiotic group	Outcomes	Reference
1	Xiaodan Chen	2024	China	a combination of Lactobacillus reuteri GL-104, Lactobacillus paracasei, Lactobacillus rhamnosus, Lactobacillus acidophilus GL-206, and Bifidobacterium longum.	2 × 10^9^ CFU/d (powdered probiotic, twice daily)	6 months	4.44 ± 0.93	4.44 ± 1.03	66	32	34	14/18	16/18	intestinal microbiota detection, serum immune index detection (IgE,IL-4, IL-13, Th1/Th2, CRP), pulmonary function test, FEV1/VC max	(26)
2	Lorenzo Drago	2022	Italy	a mixture of Ligilactobacillus salivarius LS01 (DSM 22775) and Bifidobacterium breve B632 (DSM 24706)	2 × 10^9^ CFU/d (1 × 10^9^ CFU Ligilactobacillus salivarius LS01 + 1 × 10^9^ CFU Bifidobacterium breve B632), twice daily	16 weeks	7 ± 2.95	7 ± 3.38	422	210	212	91/119	91/121	number, severity, and duration of asthma exacerbations, intensity of maintenance and as need treatments, and safety	(25)
3	Jonatas Chistian Vieira Moura	2019	Brazil	Lactobacillus reuteri DS17938	one capsule 10^8^ CFU/d	60 days	10.2 ± 2.5	11 ± 2.5	30	16	14	10/6	7/7	Asthma Control Test (ACT), spirometry to determine FEV1, PEF, and FEV1/FVC, and self-report of the symptoms they experienced associated with asthma	(24)
4	Maryam Hassanzad	2019	Iran	A synbiotic compound named Kidilact®, containg Lactobacillus Casei, Bifidobacterium infantis, Lactobacillus acidophilus, Bifidobacterium breve, Lactobacillus rhamnosus, Streptococcus thermophiles, Lactobacillus bulgaris, Fructooligosaccharide (FOS)/[Prebiotic]	1 × 10^9^ CFU/d, one sachet daily	6 months	6.6 ± 2.4	6.9 ± 2.7	81	35	46	19/16	29/17	The frequency of asthma attacks, the number of outpatient visits, and the frequency of hospitalization	(23)
5	Chian-Feng Huang	2018	Taiwan	Lactobacillus paracasei (LP), Lactobacillus fermentum (LF)	1 × 10^9^ CFU/d	3 months	7.86 ± 2.5	7.68 ± 2.21, 7.37 ± 2.34	147	35	112	18/17	65/57	Global Initiative for Asthma–based asthma severity, Childhood Asthma Control Test (C-ACT) scores, Pediatric Asthma Severity Scores, Pediatric Asthma Quality of Life Questionnaire scores, peak expiratory flow rates (PEFRs), serum immune biomarker levels, and fecal probiotic microbial composition	(22)
6	Michele Miraglia Del Giudice	2017	Italy	Bifidobacteria mixture containg B longum BB536, Binfantis M-63, and B breve M-16V	Bifidobacteria mixture, B longum BB536 (3 × 10^9^ CFU/d), Binfantis M-63 (1 × 10^9^ CFU/d), and B breve M-16V (1 × 10^9^/CFU/d) as powder in 3 mg sachet, 1 sachet daily	8 weeks		9 ± 2.2	40	20	20	9/11	9/11	Total symptom score (TSS), quality of life (QoL)	(21)
7	Joanna Jerzynska	2016	Poland	Lactobacillus rhamnosus GG	1 × 10^9^ CFU/d, one dose daily	5 months		5–12	44	24	20			a symptom-medication score, lung function, exhaled nitric oxide concentration,the immunologic efficacy measured by the following: CD4+ CD25+ Foxp3+ (forkhead box P3) cells, Toll-like receptor (TLR) 4, interleukin (IL) 1, IL-6, tumor necrosis factor, IL-10, and transforming growth factor *β*-1 levels in cell culture supernatants	(20)
8	Hamid Ahanchian	2016	Iran	a Synbiotic mixture of Lactobacillus casei, Lactobacillus rhamnosus, Streptococcus thermophilus, Bifidobacterium breve, Lactobacillus acidophilus, Bifidobacterium infantis, Lactobacillus bulgaricus, and Fructooligosacharide (Zist Takhmir, Tehran, Iran)	1 × 10^9^ CFU/d	60 days	8.2 ± 2.1	8.1 ± 1.7	72	36	36	23/13	22/14	the number of viral respiratory infections, and secondary outcomes were school absence, salbutamol and prednisolone usage, outpatient visits, and hospital admission for asthma	(19)

Notes: CFU/d, colony forming units/day.

### 3.2 Risk of bias

**Figure 2:**
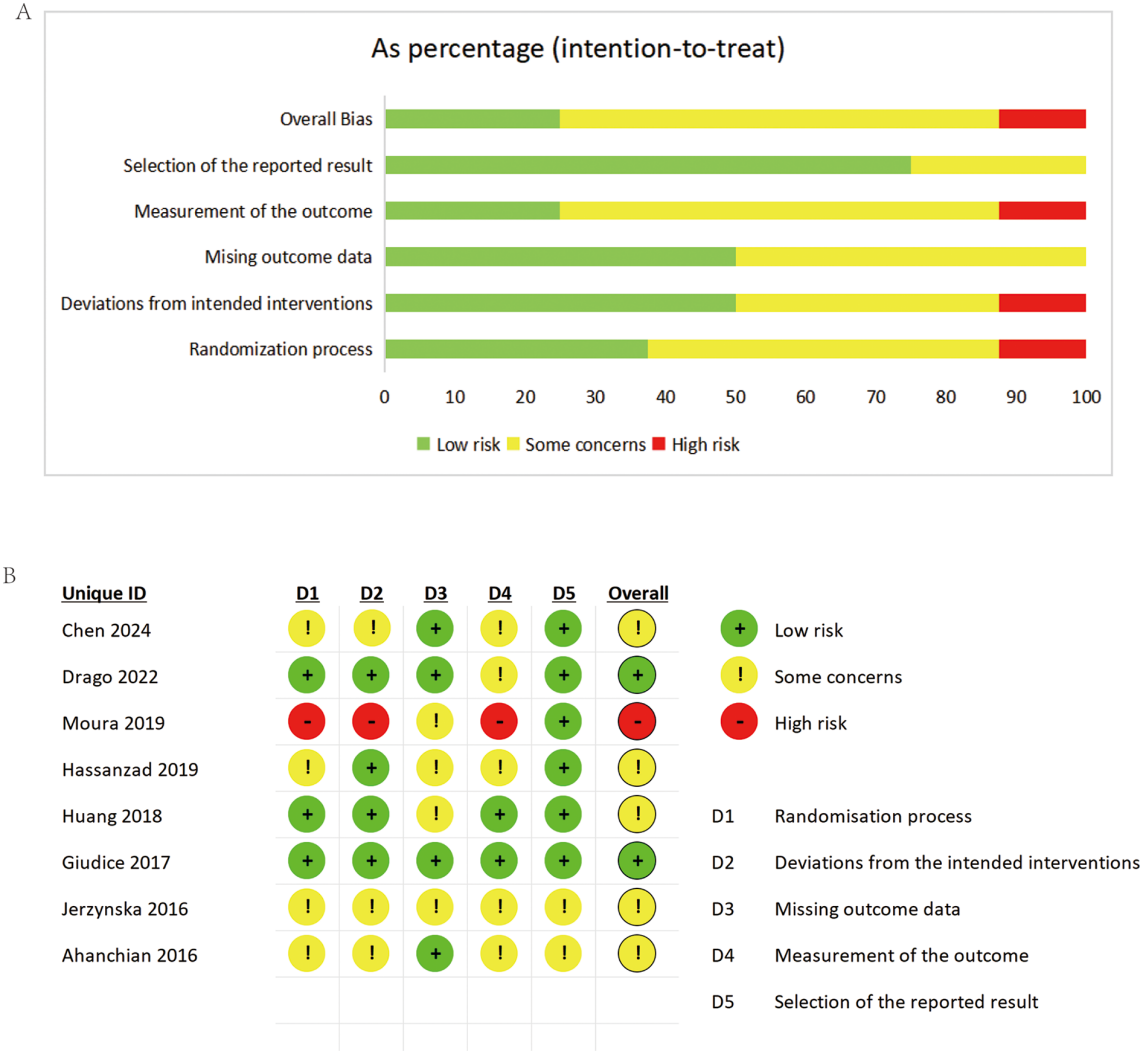
Risk of bias summary **(A)** and risk of bias graph **(B)** for included studies.

Risk of bias evaluation followed Cochrane Collaboration's guidelines. All RCTs demonstrated high methodological quality. Figure 2 present the detailed risk of bias assessment results.

### 3.3 Probiotics and the number of asthma exacerbation

**Figure 3:**
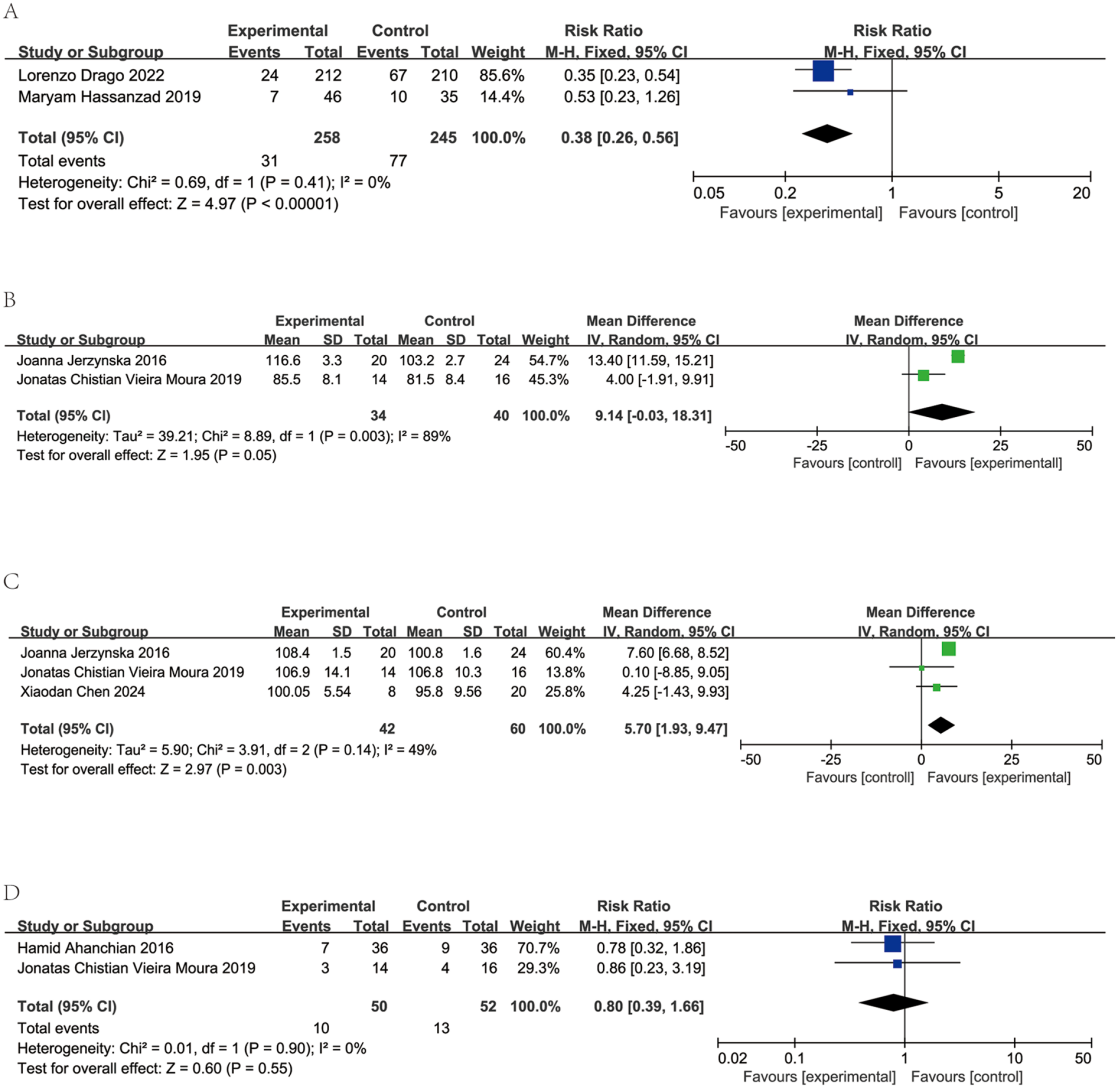
Forest plots and meta-analysis of RCTs comparing probiotics treatment vs. control group in children with asthma. **(A)** Comparison of the number of asthma exacerbations between probiotics and control group. **(B)** Comparison of the results of forced expiratory volume in the first second (FEV1) between probiotics and control group. **(C)** Comparison of the results of forced expiratory volume in the first second/forced expiratory volume (FEV1/FVC) (%) between probiotics and control group. **(D)** Comparison of the number of absent from school between probiotics and control group.

Two studies encompassing 503 participants compared acute asthma episodes between probiotics or synbiotics vs. placebos. Low heterogeneity was observed (*I*^2^ = 0%, *p* = 0.41), warranting a fixed-effects model. Meta-analysis showed significantly fewer acute episodes in the intervention group with relative risk (RR) of 0.38 [95% Confidence Interval (CI): 0.26–0.56, *p* < 0.00001]. A summary of these findings is depicted in Figure 3A.

### 3.4 Probiotics and lung function

Two studies encompassing 74 patients assessed FEV1 levels in patients receiving probiotics vs. placebos. Significant heterogeneity was detected (*I*^2^ = 89%, *p* = 0.003). Consequently, a random-effects model was employed. FEV1 showed no significant difference between groups (MD = 9.14, 95% CI: −0.03 to 18.31, *p* = 0.05). The findings are depicted in Figure 3B.

In three studies involving 102 patients, FEV1/FVC ratios were reported for those on probiotics and placebos. Low heterogeneity was observed (*I*^2^ = 49%, *p* = 0.14), supporting a random-effects mode. Data aggregation revealed a significantly higher FEV1/FVC ratios in the probiotic groups (MD = 5.70, 95% CI: 1.93–9.47, *p* < 0.003). The results are presented in Figure 3C.

### 3.5 Probiotics and absent from school

Two studies, comprising a total of 102 patients, reported number of absent from school among those administered probiotics and those given placebos. Minimal heterogeneity was detected (*I*^2^ = 0%, *p* = 0.90), warranting a fixed-effects model. Upon consolidation of the data, no significant difference in school absenteeism was observed between the probiotic and placebo groups (MD = 0.8, 95% CI: 0.39–1.66, *p* = 0.55, as shown in Figure 3D).

### 3.6 Table bias and sensitive analysis

**Figure 4:**
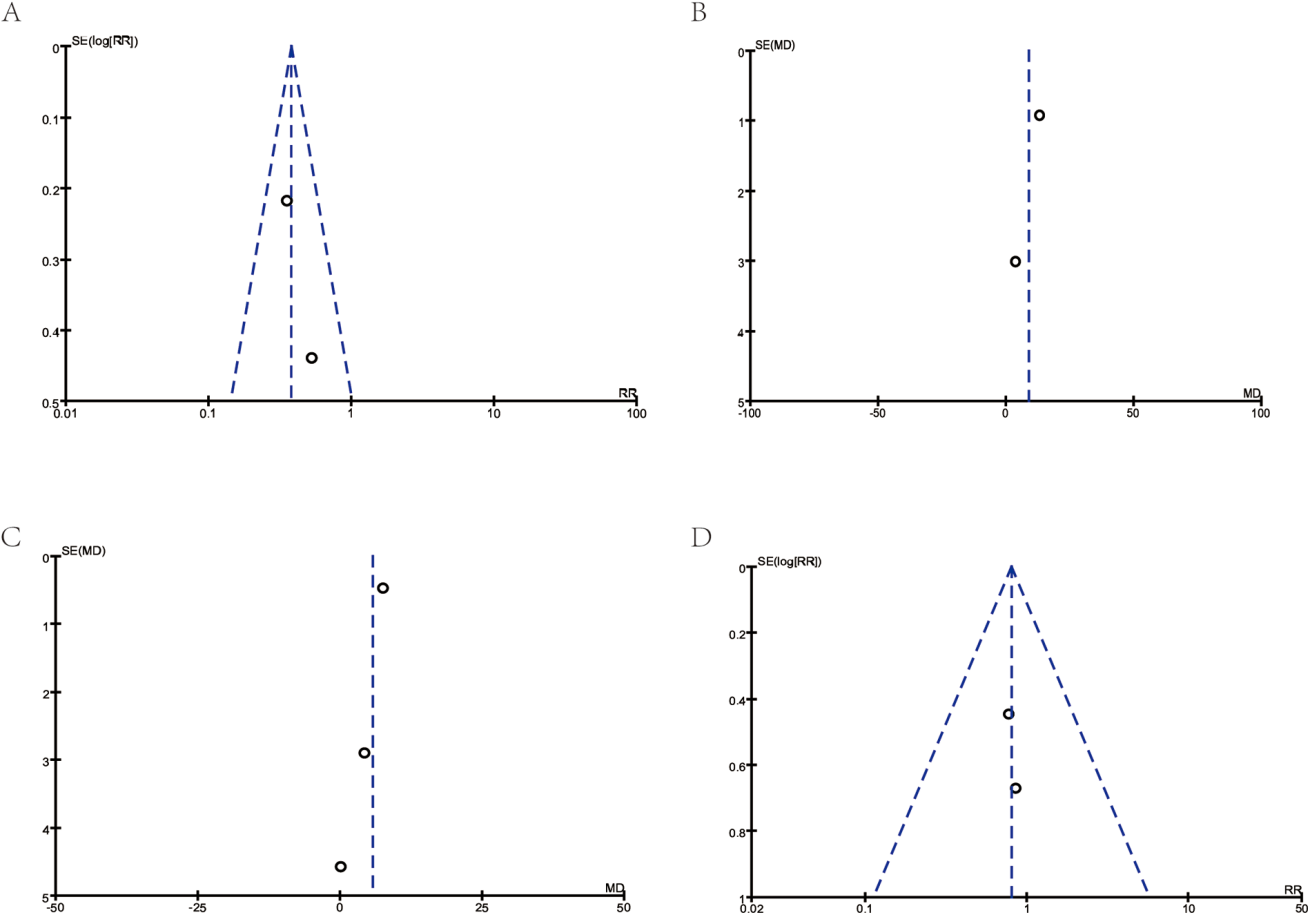
Funnel plots for publication bias. **(A)** Corresponds to Figure 3A: Asthma exacerbations. **(B)** Corresponds to Figure 3B: FEV1. **(C)** Corresponds to Figure 3C: FEV1/FVC (%). **(D)** Corresponds to Figure 3D: school absences.

Although the sample size is relatively small, the symmetrical funnel plot (Figure 4) indicates minimal publication bias, supporting the relative reliability of our findings.

## 4 Discussion

In the current study, we encompassed eight RCTs published in Science Citation Index journals from 2014 to 2024, involving 902 participants to evaluate probiotic interventions in children with asthma. The synthesized evidence demonstrated beneficial effects of probiotics, including improved asthma symptoms, reduced frequency of acute exacerbation, and enhanced in pulmonary function as measured by the FEV1/FVC ratio. These comprehensive findings indicate the potential therapeutic value of probiotics as a complementary approach in pediatric asthma management.

### 4.1 Clinical benefit of probiotics in asthma management

Asthma exacerbation refers to a sudden worsening of asthma symptoms that requires additional treatment. Managing asthma effectively aims to reduce the frequency and severity of exacerbations. By conducting the meta-analysis of two studies (*n* = 509) with very low heterogeneity (24, 25), we found that probiotics or synbiotics can significantly reduce asthma attacks in children with asthma (RR = 0.38, 95% CI: 0.26–0.56, *p* < 0.00001). These findings align with Das et al.'s 2013 meta-analysis of 12 studies (*n* = 995), which reported improved quality-of-life scores in allergic rhinitis patients and delayed asthma attack onset with probiotic supplementation (27). Additionally, Du et al' s strain-specific analysis (28) revealed that Lactobacillus rhamnosus GG supplementation during pregnancy and infancy reduced asthma occurrence (RR 0.75, 95% CI: 0.57–0.99, *I*^2^ = 11%; *p* = 0.04). Improvements in ACT were reported separately in two included studies (22, 24), one of which used estimating equation model while the other did not, so it could not be combined for meta-analysis. Nevertheless, the collective evidence suggests probiotics’ therapeutic potential in managing asthma attacks.

Pulmonary function tests serve as crucial diagnostic tolls in pediatric asthma, enabling assessment of asthma control, monitor the effectiveness of asthma treatment plans, and assist doctors in adjusting treatment regimens. Due to the particularities of children, there may be some challenges and difficulties in conducting pulmonary function tests. Our assessment and analysis of two studies (20, 24) involving a total of 74 participants found a trend towards improvement in FEV1 with probiotic supplementation, although no significant difference was observed (MD = 9.14, 95% CI: −0.03 to 18.31, *p* = 0.05). An evaluation and analysis of three studies (20, 24, 26) involving a total of 102 participants revealed that probiotic supplementation can significantly improve FEV1/FVC (MD = 5.70, 95% CI: 1.93–9.47, *p* < 0.003), suggesting a positive impact on the physiological aspects of asthma, potentially leading to better respiratory health for children.

Reports indicate that asthma imposes the heaviest disability burden on children, resulting in nearly 13.8 million school absences in the United States in 2013 (4). The assessment and analysis of two studies (19, 24) involving 102 participants included in our review showed that probiotic supplementation did not significantly differ in reducing school absences among children with asthma.

### 4.2 Safety and tolerability

The included studies consistently demonstrated a favorable safety profile and good tolerability for probiotics (as shown in Table 1). The incidence of reported adverse events, such as mild gastrointestinal discomfort (e.g., diarrhea or bloating), was low and comparable between the probiotic and placebo groups. No significant adverse effects related to probiotic use were reported, suggesting that probiotics can be a safe addition to the treatment regimen for children with asthma. For instance, Drago et al. (25) specifically monitored safety outcomes and observed no significant differences in adverse event rates between the probiotic and placebo groups. These findings are particularly relevant given the chronic nature of asthma and the critical need for safe, long-term management strategies.

### 4.3 Assessment of certainty of evidence

To evaluate the certainty of evidence, we applied the GRADE framework. Based on this evaluation, the certainty of evidence for key outcomes in this study is summarized in Table 2 as follows:

**Table 2:** GRADE assessment table.

Outcome	Sample size (*n* participants)	Study design	Risk of bias	Inconsistency	Indirectness	Imprecision	Downgrading factors	Upgrading factors	Overall quality of evidence	Notes
Asthma exacerbation rates	503	RCTs	Low	Moderate	None	None	Potential bias in randomization and allocation concealment (−1)	None	Moderate	Further research is needed to reduce the potential for bias.
FEV1/FVC ratio	102	RCTs	Low	Low	None	None	Low heterogeneity (*I*^2^ = 49%), consistent results, no downgrading	None	High	Results are stable, and the quality of evidence is high.
FEV1 Improvement	74	RCTs	Low	High	None	Moderate	High heterogeneity (*I*^2^ = 89%), small sample size, imprecise results (−2)	None	Low	Insufficient sample size leads to significant uncertainty in results.
School Absence Rate	102	RCTs	Low	High	None	High	Small sample size, wide confidence intervals (−1)	None	Moderate	Small sample size limits the reliability of the results.

Abbreviations: GRADE, grading of recommendations assessment, development and evaluation; FEV1, forced expiratory volume in 1 s; FVC, forced vital capacity; RCTs, randomized controlled trials.

Acute asthma exacerbation rates: The evidence was initially rated as high due to the inclusion of randomized controlled trials (RCTs). However, concerns regarding potential bias in randomization processes and allocation concealment led to a downgrade. The certainty of evidence for this outcome was rated as moderate.

FEV1/FVC ratio: This outcome demonstrated low heterogeneity (*I*^2^ = 49%) and consistent results across studies. The certainty of evidence was rated as high.

FEV1 improvement: Significant heterogeneity (*I*^2^ = 89%) and small sample sizes contributed to imprecision in the findings. Consequently, the certainty of evidence was downgraded to low.

School absenteeism: While heterogeneity was minimal (*I*^2^ = 0%), the small sample size and wide confidence intervals led to a downgrade. The certainty of evidence was rated as moderate.

Immune-related factors (e.g., IgE, IL-4, IL-13): Variability in detection methods and small sample sizes limited the reliability of findings, resulting in a low certainty of evidence.

This evaluation highlights the need for caution in interpreting certain outcomes, particularly those with low or moderate certainty. Future research should prioritize addressing methodological limitations to enhance the reliability of evidence.

### 4.4 Mechanisms of probiotic action

The precise mechanisms underlying probiotic-mediated effects in asthma remain not fully elucidated. Current evidence suggests multiple pathways, including immunomodulation, gut microbiota restoration, and inflammatory response attenuation. In this study, three RCTs including 257 participants were included to examine the expression of immune-related factors (20, 22, 26). Among them, Chen et al. (26) found that probiotic supplementation could downregulate IgE, IL-4, and IL-13 in children with asthma. Huang et al. (22) reported no significant change in IL-4 after probiotic supplementation. Due to the variation in detection methods used in each study, resulting in different units of measurement, a meta-analysis could not be conducted.

Emerging evidence highlights the significance of the gut-lung axis in asthma pathophysiology (29–31). Intestinal microbiota orchestrates immune system development and homeostasis, with dysbiosis implicated in asthma and other allergic disorders. The therapeutic potential of probiotics in asthma management, hypothesized to act through gut microbiota restoration and subsequent gut-lung axis modulation, warrants further investigation.

### 4.5 Limitations

While this meta-analysis provides valuable insights into the potential role of probiotics in managing pediatric asthma, several limitations of both the included studies and the meta-analysis itself must be acknowledged. These limitations could affect the robustness, generalizability, and interpretability of the findings.

#### 4.5.1 Sample size

Many of the included randomized controlled trials (RCTs) had relatively small sample sizes, particularly in subgroup analyses such as those assessing FEV1 improvements, which involved only 74 participants. The overall sample size of this meta-analysis (902 participants) may also be insufficient to represent the diverse population of children with asthma, potentially limiting the statistical power and generalizability of the conclusions.

#### 4.5.2 Heterogeneity

There was considerable heterogeneity among the included studies in terms of probiotic interventions, particularly for key outcomes such as FEV1 improvement. While a random-effects model was appropriately applied to account for this heterogeneity, further exploration of its sources is necessary. The following factors likely contributed to the observed variability:

Probiotic strains:

The included studies used a wide variety of probiotic strains, including *Lactobacillus rhamnosus, Bifidobacterium breve*, and combinations of multiple strains. Different strains may have distinct mechanisms of action, such as modulating immune responses, reducing inflammation, or restoring gut microbiota balance, leading to variability in clinical outcomes. For example, *Lactobacillus rhamnosu*s GG has shown efficacy in reducing asthma exacerbations in some studies (28), while others reported inconsistent results (32). Combined probiotic interventions generally demonstrated superior efficacy compared to single strains (33, 34), but the use of multiple strains poses challenges in understanding the specific mechanisms through which probiotics improve asthma. This strain-specific variability complicates the interpretation of pooled results.

Dosage:

The dosages of probiotics varied significantly among the included studies, ranging from 10^8^ to 10^10^ CFU/day. Higher doses may have a more pronounced therapeutic effect, but the optimal dosage for managing pediatric asthma remains unclear. The lack of dosage standardization across studies likely contributed to the heterogeneity in outcomes. For example, Lin et al. (32) reported no significant association between probiotic supplementation and asthma risk, possibly due to the wide variation in dosages used. This underscores the need for future studies to establish dose-response relationships.

Intervention durations:

The duration of probiotic supplementation also varied widely, from as short as 4 weeks to as long as 12 months. Short-term interventions may not provide sufficient time for probiotics to exert measurable effects on clinical outcomes, while longer durations may yield more substantial improvements. For instance, Du et al. (28) found that *Lactobacillus rhamnosus* GG supplementation during pregnancy and infancy reduced asthma risk, suggesting that longer interventions may have preventive effects. However, short-term studies fail to capture the long-term effects and safety of probiotic supplementation, which is particularly important given the chronic nature of asthma.

Timing of supplementation:

The timing of probiotic administration (e.g., during infancy, early childhood, or after asthma diagnosis) may also influence outcomes. Some studies suggest that early-life supplementation may have preventive effects on asthma development, while later supplementation focuses on symptom management. For example, Wei et al. (30) found no overall benefit of probiotics in reducing asthma risk but observed reduced wheezing incidence in atopic infants (RR = 0.61, 95% CI: 0.42–0.90). This timing difference could further contribute to heterogeneity in results.

Study populations:

Differences in participant characteristics, such as age, baseline asthma severity, and comorbidities, may also have influenced the observed outcomes. For example, probiotics may have a more pronounced effect in children with mild asthma compared to those with severe disease. Lin et al. (29) highlighted that variability in inclusion criteria across studies, such as asthma severity and comorbid allergic conditions, contributed to inconsistent findings in their meta-analysis. This heterogeneity underscores the need for more uniform participant selection criteria in future research.

#### 4.5.3 Geographic and methodological biases

The geographic distribution of the included studies was skewed, with most being conducted in high-income countries. This lack of representation from low- and middle-income regions, where asthma prevalence and characteristics may differ, restricts the global applicability of the findings. In addition, the studies provided limited mechanistic insights into how probiotics may benefit asthma patients. Key immune markers and gut microbiota data were inconsistently reported, preventing a deeper exploration of the pathways involved, such as immune modulation or the gut-lung axis.

Additionally, the meta-analysis only included peer-reviewed publications, excluding grey literature such as conference abstracts, dissertations, and unpublished studies. This may have introduced publication bias, particularly if studies with negative or null results were less likely to be published. Methodological biases, such as inconsistencies in randomization and allocation concealment, could also affect the reliability of the findings.

#### 4.5.4 Scope of outcomes

This meta-analysis primarily focused on clinical outcomes, such as asthma exacerbation rates and lung function. Other important outcomes, such as quality of life and healthcare resource utilization, were not systematically assessed due to insufficient data. While two studies reported data on quality of life, the information was inadequate for statistical analysis. Future research should prioritize these broader outcomes to provide a more comprehensive understanding of the impact of probiotics on pediatric asthma management.

### 4.6 Implication for future research

To address these limitations, future studies should prioritize large-scale, multicenter RCTs with sufficient sample sizes to improve statistical power and representativeness. Standardization of probiotic interventions, including strain selection, dosage, and duration, is essential to reduce heterogeneity and enhance comparability across studies. Longer follow-up periods should also be incorporated to assess the sustained effects and safety of probiotics in managing asthma as a chronic condition.

Efforts should be made to include studies from diverse geographic regions, particularly low- and middle-income countries, to improve the global applicability of findings. Mechanistic studies, integrated with clinical trials, should explore immune modulation, gut microbiota changes, and address key sources of variability, such as differences in probiotic strains, dosages, and intervention durations, using standardized methods for reporting immune-related markers. Specifically, future investigations should prioritize elucidating the mechanistic pathways, particularly probiotic-immune interactions and gut-lung axis modulation. Understanding how probiotics influence the gut-lung axis could provide crucial insights into their role in asthma management and pave the way for targeted interventions.

By addressing these limitations and focusing on mechanistic pathways like the gut-lung axis, future research can provide stronger evidence for the role of probiotics in pediatric asthma management, ultimately guiding clinical practice and improving patient outcomes.

## 5 Conclusion

This systematic review and meta-analysis demonstrate the potential of probiotics as a safe and effective complementary therapy for pediatric asthma. Probiotic supplementation significantly reduced asthma exacerbations and improved pulmonary function, particularly FEV1/FVC ratios. However, variability in probiotic strains, dosages, and intervention durations, as well as limited geographic diversity among studies, highlights the need for further high-quality research. Future studies should focus on standardizing probiotic interventions, exploring underlying mechanisms such as the gut-lung axis, and expanding research to underrepresented regions to enhance global applicability. While promising, probiotics require more robust evidence to establish their role in routine clinical practice for managing pediatric asthma.

## Data Availability

The original contributions presented in the study are included in the article/Supplementary Material, further inquiries can be directed to the corresponding authors.

## Supplementary material

Supplementary File S1 Literature search strategy.
