# Supplementary material for: Probiotics for children with asthma: a systematic review and meta-analysis
Source: Front Pediatr. 2025 Apr 24;13:1577152. doi: 10.3389/fped.2025.1577152 (PMC12058802; doi:10.3389/fped.2025.1577152)
Supplement: Supplementary File S1 — Literature search strategy. [file Supplementaryfile1.docx]

**Supplementary file 1**

**Search Strategy**

*The search formats were modified according to the search formula specifications of different databases.

For Pubmed, the search terms are as the following:

((((("Asthma"[Mesh]) OR (Asthmas[Title/Abstract])) OR (Asthma, Bronchial[Title/Abstract])) OR (Bronchial Asthma[Title/Abstract])) AND (((((("Asthma"[Mesh]) OR (Asthmas[Title/Abstract])) OR (Asthma, Bronchial[Title/Abstract])) OR (Bronchial Asthma[Title/Abstract])) AND ((((((((((((((((("Probiotics"[Mesh]) OR (Synbiotics[Title/Abstract])) OR (Lactobacillales[Title/Abstract])) OR (Bifidobacterium[Title/Abstract])) OR (saccharomyces[Title/Abstract])) OR (Streptococcus[Title/Abstract])) OR (LGG[Title/Abstract])) OR (BB12[Title/Abstract])) OR (BB-12[Title/Abstract])) OR (Helveticus[Title/Abstract])) OR (Reuteri[Title/Abstract])) OR (Paracasei[Title/Abstract])) OR (Lactis[Title/Abstract])) OR (Boulardii[Title/Abstract])) OR (Bifidum[Title/Abstract])) OR (Rhamnosus[Title/Abstract])) OR (acidophilus[Title/Abstract]))) AND ((("Child"[Mesh]) OR (Children[Title/Abstract])) OR (infant[Title/Abstract])))) AND ((((((("Child"[Mesh]) OR (Children[Title/Abstract])) OR (infant[Title/Abstract])) OR (Pediatric[Title/Abstract])) OR (paediatric[Title/Abstract])) OR (Adolescence[Title/Abstract])) OR (adolescent[Title/Abstract]))
